# ITGA5 is overexpressed and promotes tumor progression through SNAI2 in OSCC

**DOI:** 10.3389/fcell.2026.1685462

**Published:** 2026-05-21

**Authors:** Yanan Li, Li Kong, Kai Zhou, Jinfei Gu, Liping Sun, Shuxin Ding, Bo Zou, Dongping Zhang, Zhen Meng

**Affiliations:** 1 Biomedical Laboratory, Medical School of Liaocheng University, Liaocheng, China; 2 Department of Oral and Maxillofacial Surgery, Jinan Stomatology Hospital, Jinan, China; 3 Department of Stomatology, The Second People’s Hospital of Zhangdian District, Zibo, China; 4 Department of Stomatology of Liaocheng People’s Hospital, Liaocheng, China

**Keywords:** bioinformatics, cell proliferation, integrin subunit α 5, migration and invasion, oral squamous cell carcinoma, snail family transcriptional repressor 2

## Abstract

Integrin subunit α5 (ITGA5) has been recognized as a potential diagnostic biomarker across multiple cancer types. ITGA5 is upregulated in head and neck squamous cell carcinoma (HNSCC). Oral squamous cell carcinoma (OSCC), with poor prognosis and high mortality, accounts for two-thirds of HNSCC cases. However, the biological functions and underlying mechanisms of ITGA5 in OSCC progression remain unclear. In the present study, we analyzed the expression, prognostic significance, function, and co-expression genes of ITGA5 in HNSCC using a series of public biological information databases, detected the role of ITGA5 in OSCC progression, and discussed the underlying mechanisms. The results indicate that both the mRNA and protein level of ITGA5 were upregulated and related to the prognosis of HNSCC. Knockdown of ITGA5 inhibited cell proliferation, invasion, migration, and epithelial-mesenchymal transition (EMT). However, overexpression of snail family transcriptional repressor 2 (SNAI2) reversed the function of ITGA5 in regulating OSCC cell proliferation, invasion, migration, and EMT. In conclusion, our study suggests that ITGA5 is upregulated in HNSCC and associated with poor prognosis; functional studies indicate ITGA5 promotes OSCC proliferation and metastasis via SNAI2, indicating that ITGA5 may be a potential therapeutic target for OSCC.

## 1 Introduction

Head and neck squamous cell carcinoma (HNSCC), originating from the mucosal epithelium of the oral cavity, pharynx and larynx, is the most common malignant tumor in the head and neck region (Johnson et al., 2020). Oral squamous cell carcinoma (OSCC) is the most common type of HNSCC, with about 370,000 new cases and 177,000 deaths in 2020 (Sung et al., 2021). OSCC is characterized by high morbidity and mortality. The 5-year survival rate of OSCC is about 60%, but it varies from 10% to 82% depending on stage, age, race, comorbidity, and location in the oral cavity (Chinn and Myers, 2015). Smoking, alcohol abuse, areca nut chewing and human papillomavirus (HPV) infection are the main risk factors for OSCC in China (Zhang et al., 2018). In recent years, significant progress has been made in cancer treatment methods, including surgery, chemotherapy, and radiotherapy, as well as targeted therapy and immunotherapy (Cramer et al., 2019; Ren et al., 2017; Yang et al., 2019). So far, surgery combined with adjuvant radiotherapy is the standard treatment for locally advanced OSCC (Shah et al., 2023); however, surgery often leads to facial tissue defects or functional impairments, and radiation resistance and tissue damage are obstacles to the use of radiotherapy. Chemotherapy is one of the important treatment methods for patients with metastatic tumors; Nevertheless, patients also face a greater risk of oral side effects from chemotherapy, such as slowed tissue healing, bone and salivary gland damage and disintegration, and disruption of the normal bacterial balance in the mouth (Shah et al., 2023). Emerging approaches such as targeted therapy and immunotherapy have opened up new directions for OSCC treatment. However, challenges such as metastasis, recurrence, and therapeutic resistance are still unsolved. Therefore, elucidating the pathogenesis and exploring new biomarkers and treatment strategies are in urgent need for patients with OSCC.

Integrins, including several cell membrane adhesion molecules, mediate cell-cell and cell-matrix interactions (Maltseva et al., 2025). Integrins play crucial roles in several pathological and physiological processes based on their attachment to the extracellular matrix (ECM), which directly controls cell migration and invasion (Guo and Giancotti, 2004). Integrins consist of α and β subunits. Integrin subunit α5 (ITGA5) often binds to integrin subunit beta 1 (ITGβ1) to form a heterodimer called the fibronectin receptor (Morgan et al., 2009), which is closely associated with cell proliferation, angiogenesis, tumor metastasis, and carcinogenesis (Deng et al., 2019; Hou et al., 2020; Lee et al., 2009; Li X. et al., 2020). It is reported that ITGA5 is the key subunit responsible for promoting the proliferation, migration and invasion of OSCC (Deng et al., 2019). Through bioinformatics analysis, we previously found that ITGA5 is overexpressed in OSCC and associated with survival and node metastasis, indicating that ITGA5 can be a potential diagnostic biomarker and therapeutic targets; however, the underlying mechanism remains unclear.

SNAI2, also known as Slug, is a prototypical epithelial-to-mesenchymal transition transcriptional (EMT) factor belonging to the Snail family of zinc-finger transcription factors (Barrallo-Gimeno and Nieto, 2005; Thiery, 2002). SNAI2 is associated with a wide variety of biological processes in tumor progression, including tumor growth, metastasis, cellular differentiation, angiogenesis, immune escape, and DNA damage repair (Barrallo-Gimeno and Nieto, 2005; Gross et al., 2019; Hensch et al., 2023; Hultgren et al., 2020; Meng et al., 2024). SNAI2 is highly expressed in OSCC cells, and knockdown of SNAI2 represses anti-tumor drug resistance and the invasive properties of OSCC (Nakamura et al., 2018); through transcriptional activation of SNAI2, FOXD1 promotes EMT in OSCC (Chen et al., 2021); in addition, TGF-β1 regulates cell invasion through regulating SNAI2 in OSCC (Joseph et al., 2009). These studies indicate the importance of SNAI2 in OSCC progression. However, the role and regulation of SNAI2 in OSCC are still not fully understood.

In this study, a series of open-access databases were used to analyze the expression and prognostic value of ITGA5 in patients with HNSCC. In addition, we detected the biological function of ITGA5 in OSCC and revealed that ITGA5 regulates OSCC progression through SNAI2, suggesting that the ITGA5-SNAI2 axis may be a diagnostic and therapeutic target for OSCC.

## 2 Materials and methods

### 2.1 TIMER database

The Tumor Immune Estimation Resource (TIMER) (http://timer.cistrome.org/) web server is a comprehensive resource for systematic analysis across diverse cancer types (Li T. et al., 2020). The Diff Exp module of TIMER allows exploring differential gene expression between tumor and adjacent normal tissues. In our study, using the Diff Exp module, we input “GENE: ITGA5” to identify the expression that is upregulated or downregulated in the tumors compared to normal tissues. The statistical significance was computed by the Wilcoxon test.

### 2.2 UALCAN database

The University of ALabama at Birmingham CANcer data analysis Portal (UALCAN) database (https://ualcan.path.uab.edu/) is a comprehensive and interactive web resource for analyzing cancer OMICS data (Chandrashekar et al., 2022). UALCAN provides easy access to publicly available cancer OMICS data (TCGA, MET500, CPTAC and CBTTC). The Clinical Proteomic Tumor Analysis Consortium (CPTAC) data were used to analyze protein expression of ITGA5 in normal tissues, cancer tissues, and the tumor grade of HNSCC. Inclusion criteria: samples with complete clinical annotation and pathologically confirmed primary HNSCC originating from oral cavity sites (including hard palate, gums, tongue, floor of the mouth, retromolar pad, buccal mucosa). Exclusion criteria: samples without clinical information, non-oral HNSCC subtypes, recurrent or metastatic tumors. After filtering, a final cohort of 362 primary tumor samples and 32 matched normal adjacent tissues was included for subsequent analysis.

### 2.3 Human protein atlas database

The Human Protein Atlas (HPA) (https://www.proteinatlas.org/) is a public database storing millions of microscopic images of proteins of various human tissues and cells (Thul et al., 2017). To analyze the expression of ITGA5 using antibodies (CAB009008, HPA002642) in normal tissues and cancer tissues of HNSCC, we entered the gene “ITGA5” in the search box of the HPA database. Then, according to prognostic summary, we searched for association between high expression of ITGA5 gene and prognosis in all cancers.

### 2.4 Kaplan-Meier plotter database

The Kaplan-Meier plotter (https://kmplot.com/analysis/) is capable of exploring the correlation the expression of a gene (mRNA, miRNA, protein) with patients’ survival of 21 tumor types (Gyorffy, 2024). Sources for the databases include GEO, EGA, and TCGA. The screening method of this study: “Cancer: Head neck squamous cell carcinoma”; “Gene: ITGA5; SNAI2”; “Survival: overall survival (OS)”. The extracted information was used to analyze the relationship between genes and tumor prognosis.

### 2.5 Linked omics database

The Linked Omics database (http://www.linkedomics.org/) is a publicly available portal that includes multi-omics data from all 32 TCGA cancer types (Vasaikar et al., 2018). The web application provides three analytical modules: Link Finder, Link Interpreter and Link Compare. Link Finder module was used to screen ITGA5-related differentially expressed genes in the TCGA HNSC cohort (n = 503). Pearson correlation coefficient was used to perform statistical analysis. The results were presented in the form of volcanic maps, heat maps or scatter plots. The top 100 genes positively correlated with ITGA5 were analyzed by GO/KEGG pathway visualization. GO/KEGG pathway analyses were performed using R software (version 4.5.1) clusterProfiler package. The resulting q-values were all below 0.05.

### 2.6 GSEA database

The GSEA database (https://www.gsea-msigdb.org/) provides an analysis software GSEA and a set of genomic MSigDB database (Subramanian et al., 2005). We inserted the “HALLMARK_EPITHELIAL_MESENCHYMAL_TRANSITION” gene set and then downloaded a total of 200 EMT- related genes.

### 2.7 Cell culture

Human OSCC cell lines CAL27 and SCC-15 were purchased from National Biomedical Cell Resource Bank of China (BMCR), (Wuhan, Hubei, China). The cells were cultured in Dulbecco’s modified Eagle’s medium (DMEM) (Sigma, MO, USA) supplemented with 10% fetal bovine serum (FBS) (Clark, China), and placed in an incubator (D165H, RWD, Shenzhen, China) containing 5% CO_2_ at 37 °C.

### 2.8 Cell lentiviral infection experiment

The lentivirus used for ITGA5 knockdown in this study was purchased from Genechem Co., Ltd (Shanghai, China). The targeting sequence of lentivirus for knocking down ITGA5 was: CCA​CTG​TGG​ATC​ATC​ATC​CTA; the scramble control (shCon) sequence was: TTC​TCC​GAA​CGT​GTC​ACG​T. For SNAI2 overexpression, the full-length human SNAI2 cDNA (NCBI Gene ID: 6591) was cloned into the pHBLV-CMV-MCS-IRES-Puro (Chemicalbook; Beijing Xilin Buke Network Technology Co., Ltd.). For the control, the empty vector was used. Lentiviruses were produced by co-transfecting the recombinant plasmid (or the empty vector) with the packaging plasmids psPAX2 and pMD2.G into HEK293T cells using lipofectamine 2000 (Thermofisher, MA, USA). Viral supernatants were collected at 48 and 72 h post-transfection.

Cells were incubated in 12-well plates, and when the cell confluence reached 20%–30%, 3 μL lentivirus (MOI: 10) (the MOI was determined according to the instruction manual) and 20 μL Hitrans GP (Genechem, Shanghai, China) were mixed and added to the wells. After a 15-h incubation, the cell culture medium containing lentivirus was replaced with fresh culture medium. The cells were cultured for 2 weeks in the culture medium containing 2 μg/mL purinomycin (Beyotime, Beijing, China). Cells infected with lentivirus were observed under an inverted fluorescent microscope (Olympus, Tokyo, Japan). The expression of the targeted gene was measured using quantitative real-time PCR (qRT-PCR).

### 2.9 qRT-PCR

RNA was extracted with TRIzol reagent (Invitrogen, CA, USA) according to the manufacturer’s instructions. The RNA was then reverse-transcribed into cDNA with a BeyoRT^TM^II First Strand cDNA Synthesis Kit (Beyotime, Beijing, China). qRT-PCR was performed by SYBR Green method in an Applied Biosystems 7500 machine (ABI, CT, USA). The following thermocycling conditions were used for qRT-PCR: 1) 95 °C, 10 min; 2) (58 °C, 15 s) × 40 cycles; 3) 72 °C, 30 s. All reactions were repeated in three independent experiments. β-actin was used as the internal control. The expression of genes was normalized to the internal controls using the relative quantification (2^−ΔΔCT^) method. All reactions were performed in 3 biological replicates. The primers were listed in Supplementary Table S1. Melt curve profiles were generated for all qRT-PCR reactions, confirming a single, sharp melting peak for each amplicon. This result indicates high primer specificity with no non-specific amplification or primer dimers.

### 2.10 Western blot

Proteins were extracted using RIPA reagent (#P0013B; Beyotime). The protein concentration was determined using a BCA Kit (#P0010S; Beyotime). Equal amounts of protein (30 μg per lane) were separated by 10% SDS-PAGE and transferred to PVDF membranes. After being blocked in 5% non-fat milk in TBST at 25 °C for 1 h, the membrane was incubated with primary antibodies against ITGA5 (1:1000 diluted in TBST) (#98204; Cell Signaling Technology, Inc., MA, USA), and β-actin (1:1000 diluted in TBST) (#sc-58673; Santa Cruz Biotechnology, Inc., TX, USA) for 12 h at 4 °C, and then incubated with an anti-rabbit secondary antibody (DyLight 800 4X PEG Conjugate) (1:1000 diluted in TBST) (#5257; Cell Signaling Technology, Inc.), or anti-mouse secondary antibody (DyLight 680 Conjugate) (1:1000 diluted in TBST) (#5366; Cell Signaling Technology, Inc.) at 25 °C for 1 h. The protein bands were visualized using an Amersham Typhoon Near-infrared Laser Imager imaging system (Amersham Typhoon, MI, USA). Band intensities were quantified using ImageJ software, normalized to β-actin (loading control for all blots). Quantification was performed on 3 independent biological replicates.

### 2.11 CCK-8 assay

Cells were trypsinized and prepared into cell suspension; then the cells were seeded into a 96-well plate at 6 × 10^3^ cells/well. After the cells were cultured for 0, 24, 48, and 72 h, 10 µL of CCK-8 reagent was added to each well and incubated for 3 h at 37 °C in the dark. Finally, the optical density at 450 nm was measured using a microplate reader (Thermo). The CCK-8 assay was performed on 3 independent biological replicates.

### 2.12 Colony formation assay

Cells were seeded in a 6-well plate at a density of 1 × 10^3^ cells/well and cultured for 2 weeks. The incubation was stopped and the medium was discarded when the colonies could be observed by the naked eye. Then the cells were washed three times with phosphate buffered solution (PBS) (Beyotime), and stained with 0.1% crystal violet solution (Beyotime) for 5 min at 25 °C.The cell colonies were counted using ImageJ software. This assay was performed on 3 independent biological replicates.

### 2.13 Wound scratch assay

To eliminate the confounding effect of cell proliferation on wound closure, cells were starved in serum-free DMEM for 24 h prior to scratch wounding. Cells were inoculated in 6-well plates with serum-free DMEM. Linear scratch wounds were created by a sterile 200 μL pipette tip in the cell monolayer. The detached cells were removed by washing with PBS. Cells were cultured in serum-free DMEM. At 0 h and 48 h after wounding, the wounds were imaged under a microscope (Olympus) ×40 magnification. and measured by ImageJ software. This assay was performed on 3 independent biological replicates.

### 2.14 Transwell assays

The Transwell inserts used in both migration and invasion assays had a 8-μm pore size. For transwell invasion assay, upper chambers were pre-coated with a thin layer of Matrigel (Sigma, MI, USA) at a dilution of 1:8 in serum-free DMEM and incubated at 37 °C for 6 h to allow gelation according to the manufacturer’s instructions. 200,000 cells suspended in 200 μL serum-free medium were plated into the upper chamber; whereas 800 μL DMEM with 10% FBS was added into the lower chamber as gradient. After incubation for 24 h, non-migratory cells remaining in the upper membrane surface were carefully removed and the cells on the other side of the membrane were stained with 0.1% crystal violet for 5 min. The cells were observed under a microscope ×200 magnification. Cell counting was performed in a double-blinded manner—one researcher responsible for randomly selecting 5 fields of view per sample, and another researcher counted the number of cells without knowledge of the group allocation. The cell migration assay followed similar procedures to the cell invasion assay; except that the upper chamber was non-coated and the cell seeding number was 100,000. This assay was performed on 3 independent biological replicates.

### 2.15 SNAI2 promoter-reporter construction and luciferase activity assay

The sequence of SNAI2 promoter region was obtained from Genbank (https://www.ncbi.nlm.nih.gov/gene/6591). The promoter was synthesized by Sangon Biotech (Shanghai, China), and cut by SmaI and HindIII restriction endonucleases (NEB, MA, USA). After cutting, the fregment was inserted into a pGL3-basic vector (Promega, WI, USA) digested by SmaI and HindIII enzymes, being named pGL3-SNAI2. 500ng of pGL3-SNAI2 reporter was transfected into CAL27 and SCC-15 cells by X-tremeGENE 9 DNA Transfection Reagent (Roche, Basel, Swiss). 48 h after the transfection, the cells were collected and the reporter activity was measured using a Steady-Lumi^TM^ II Firefly Luciferase Reporter Gene Assay Kit (Beyotime, Beijing, China).

### 2.16 Nude mouse tumorigenicity assay

The experiment was approved by the Ethics Committee of Liaocheng University (AP2026031355). Female BALB/c nude mice (4–6 weeks old) were used. CAL27 cells were cultured in DMEM supplemented with 10% fetal bovine serum at 37 °C in a 5% CO_2_ atmosphere. Upon reaching 80%–90% confluence, the cells were harvested, and resuspended in PBS at a concentration of 10 × 10^6^ cells/mL. For each mouse, 100 μL of the cell suspension was injected subcutaneously into the right flank under isoflurane anesthesia. Tumor growth was monitored by measuring tumor volume using the formula: volume (mm^3^) = (length × width^2^)/2. Mice were euthanized 3 weeks after injection, and the tumors were excised and weighed.

### 2.17 Statistical analysis

GraphPad Prism 8.0 (La Jolla, USA) software was employed for statistical analysis. Data from RT-qPCR, Western blot, Transwell assay, Wound scratch assay, and CCK-8 assay were expressed as mean ± standard deviation (SD). Normality was tested by Shapiro-Wilk test; all data used in parametric tests followed a normal distribution. Each independent experiment included 3 biological replicates and 3 technical replicates. Comparisons between two groups were performed using paired t-test or unpaired t-test. Comparisons among multiple groups were analyzed by two-way ANOVA. Multiple testing correction was applied using the Benjamini–Hochberg false discovery rate (FDR) method for gene correlation, GO/KEGG enrichment, and multiple-group comparisons to control Type I error. Survival analysis was performed using Kaplan-Meier method with log-rank test; prognostic factors were identified by univariate and multivariate Cox proportional hazards regression models, with the proportional hazards assumption verified by Schoenfeld residuals. *P* < 0.05 was considered to be statistically significant.

## 3 Results

### 3.1 ITGA5 expression in pan-cancer and HNSCC

**FIGURE 1:**
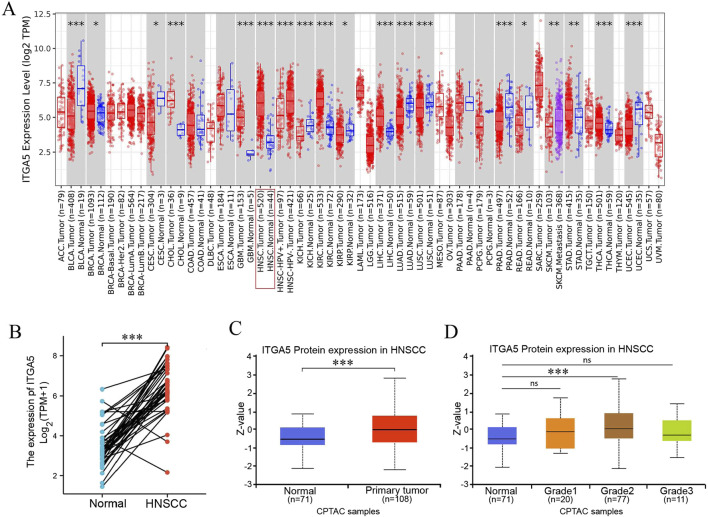
ITGA5 expression is elevated in HNSCC. **(A)** The level of expression of ITGA5 in Pan-cancer from TCGA database. **(B)** The paired differential expression analysis of ITGA5 in HNSCC and normal tissues using TCGA. **(C,D)** Differential expression of ITGA5 protein in cancer tissue and normal tissue **(C)** and tumour grade **(D)**. (*P < 0.05; **P < 0.01; ***P < 0.001).

Using the TIMER database, we analyzed the expression of the ITGA5 gene in 34 tumor types of TCGA. Figure 1 shows the mRNA expression of ITGA5 in different cancer types: the ITGA5 expression was significantly higher in tumor tissues than in adjacent normal tissues in BRCA, CHOL, GBM, HNSC, KIRC, LIHC, STAD, and THCA; whereas the ITGA5 expression was significantly lower in tumor tissues than in adjacent normal tissues in BLCA, CESC, KICH, KIRP, LUAD, LUSC, PRAD, PEAD, and UCEC (Figure 1A). The ggplot2 software package and Wilcoxon signed-rank test were used to examine the differences of ITGA5 gene expression between the tumors and adjacent normal tissues, and the results indicate that ITGA5 expression in HNSC cancer tissue samples is higher than that in normal tissue samples (Figure 1B). Moreover, based on CPTAC database, ITGA5 protein is highly expressed in HNSC tissues compared with that in normal tissues (Figure 1C); besides, there is a trends that high grades tumor possessed high TCGA protein levels (Figure 1D).

### 3.2 Prognostic analysis of ITGA5 in HNSCC

**FIGURE 2:**
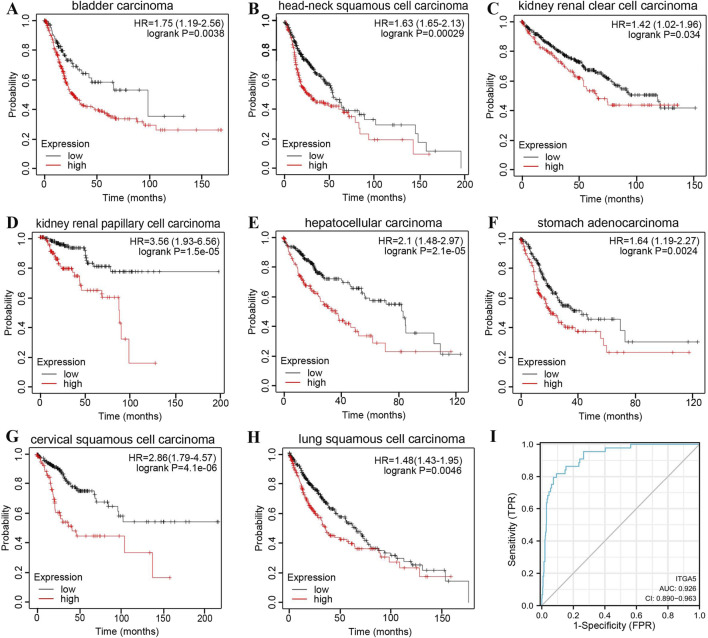
ITGA5 as a prognostic marker for multiple cancers from the HPA database. OS of patients with bladder carcinoma **(A)**, head and neck squamous cell carcinoma **(B)**, kidney renal clear cell carcinoma **(C)**, kidney renal papillary cell carcinoma **(D)**, hepatocellular carcinoma **(E)**, stomach adenocarcinoma **(F)**, cervical squamous cell carcinoma **(G)**, and lung squamous cell carcinoma **(H)** between the high-low expression groups of ITGA5 gene. **(I)** ROC curve of ITGA5.

In addition, we analyzed the association of ITGA5 expression with the survival of patients with tumors, to assess the possibility of ITGA5 as a prognostic biomarker for these patients. As shown in Figures 2A–H, high ITGA5 expression is associated with poor survival in patients with bladder carcinoma, HNSCC, kidney renal clear cell carcinoma, kidney renal papillary cell carcinoma, hepatocellular carcinoma, stomach adenocarcinoma, cervical squamous cell carcinoma, and lung squamous cell carcinoma, indicating that ITGA5 can serve as a prognostic marker in these cancers. The diagnostic value of ITGA5 upregulation for HNSCC was also confirmed by ROC curves (AUC = 0.926, 95% CI: 0.892–0.959) based on the TCGA HNSCC dataset (n = 503) (Figure 2I).

### 3.3 Prognostic value and functions of ITGA5 in regulating cell proliferation, migration, and invasion of OSCC

**FIGURE 3:**
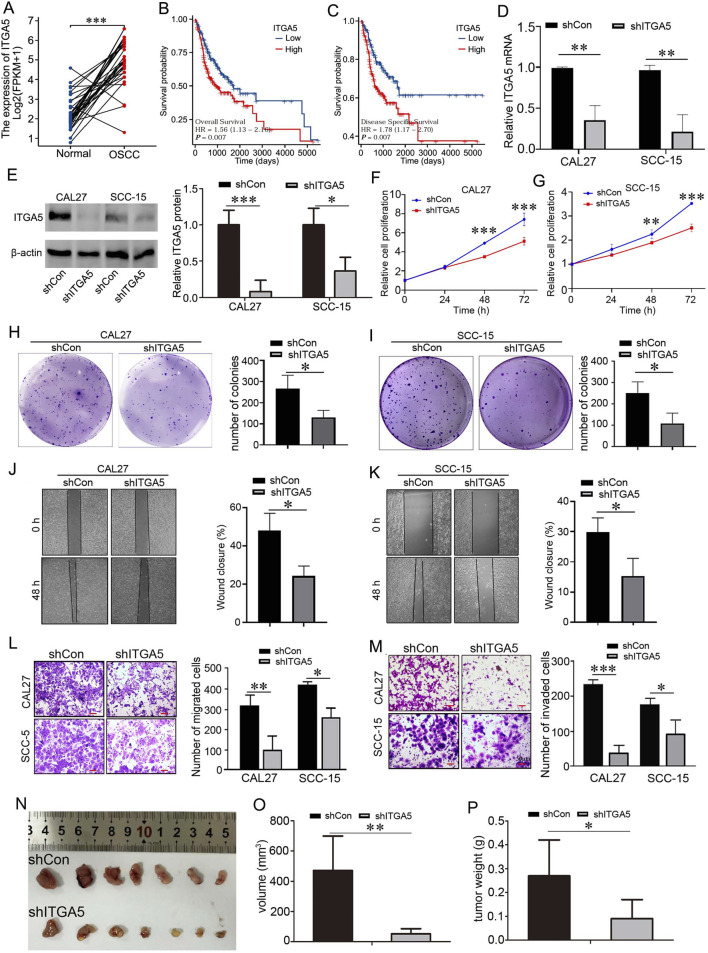
Prognostic value and functions of ITGA5 in regulating cell proliferation, migration, and invasion of OSCC. **(A)** The level of expression of ITGA5 in OSCC from TCGA database. **(B)** Overall survival of patients with OSCC. **(C)** Disease specific survival of Patients with OSCC. **(D)** ITGA5 mRNA were detected using qRT-PCR in CAL27 and SCC5 cells with Lv-shCon or Lv-shITGA5 infection. **P < 0.01. **(E)** ITGA5protein were detected using qRT-PCR in CAL27 and SCC5 cells with Lv-shCon or Lv-shITGA5 infection. *P < 0.05, ***P < 0.001. **(F,G)** Proliferation of CAL27 and SCC-15 cells with Lv-shCon or Lv-shITGA5 infection were detected using CCK-8 assay. **P < 0.01, ***P < 0.001. **(H,I)** Cell clone formation of CAL-27 cells and SCC-15 cells with Lv-shCon or Lv-shITGA5 infection. The percentage of wound closure (right panel). *P < 0.05. Bar = 100 μm. **(J,K)** Wound healing experiment was used to detect cell migration ability in CAL27 cells and SCC-15 cells with Lv-shCon or Lv-shITGA5 infection. The percentage of wound closure (right panel). *P < 0.05. Bar = 100 μm. **(L)** Transwell assay was used to detect cell migration ability in CAL27 cells and SCC-15 cells with Lv-shCon or Lv-shITGA5 infection. Number of migrated cells (right panel). Bar = 50 μm. *P < 0.05, **P < 0.01. **(M)** Transwell assay was used to detect cell invasion ability in CAL27 cells and SCC-15 cells with Lv-shCon or Lv-shITGA5 infection. Number of invaded cells (right panel). Bar = 50 μm. *P < 0.05, ***P < 0.001. **(N)** Tumor formation in nude mice. **(O)** Tumor volume. **P < 0.01. **(P)** Tumor weight. *P < 0.05.

Considering that OSCC is a main type of HNSCC, we analyzed the expression of ITGA5 in OSCC and the association of this gene with patient survival using bioinformatic methods. As shown in Figure 3A, ITGA5 was overexpressed in OSCC tissues compared with normal tissues. For the survival and Cox analyses: A total of 329 patients were included for overall survival analysis, and 312 patients for disease-specific survival analysis with complete clinical and follow-up data. Figure 3B indicate that the group of low expression of ITGA5 had better overall survival probability than the group of high expression of ITGA5. Multivariate Cox regression analysis (Table 1) showed age >60 years (HR = 1.550, 95% CI:1.096–2.192, *P* = 0.013), pathologic stage III&IV (HR = 2.172, 95% CI:1.370–3.445, *P* < 0.001), and high ITGA5 expression (HR = 1.541, 95% CI:1.090–2.176, *P* = 0.014) were independent poor prognostic factors; gender, clinical stage, and histologic grade were not significantly associated with OS (all *P* > 0.05). Univariate analysis confirmed pathologic stage III&IV (HR = 2.161, *P* < 0.001) and high ITGA5 (HR = 1.563, *P* = 0.007) as risk factors, while age >60 approached significance (*P* = 0.085). Besides, low expression of ITGA5 indicates good disease specific survival compared with high expression of this gene (Figure 3C). Multivariate Cox regression analysis (Table 2) explored disease-specific survival (DSS) risk factors in 312 patients. Pathologic stage III&IV was an independent poor prognostic factor (HR = 2.528, 95% CI: 1.332–4.799, *P* = 0.005). High ITGA5 expression approached significance (HR = 1.483, 95% CI: 0.958–2.297, *P* = 0.077). Gender, age, clinical stage, and histologic grade showed no significant associations with DSS (all *P* > 0.05). Univariate analysis confirmed pathologic stage III&IV (HR = 2.641, *P* = 0.003) and high ITGA5 (HR = 1.779, *P* = 0.007) as risk factors for poorer DSS.

**TABLE 1:** Multivariate Cox regression analysis of risk factors for overall survival.

Characteristics	Total (N)	Univariate analysis		Multivariate analysis	
		Hazard ratio (95% CI)	P value	Hazard ratio (95% CI)	P value
Gender	329	​	​	​	​
Female	102	Reference	​	​	​
Male	227	0.903 (0.645–1.266)	0.555	​	​
Age	329	​	​	​	​
≤ 60	156	Reference	​	Reference	​
>60	173	1.330 (0.961–1.840)	0.085	1.550 (1.096–2.192)	**0.013**
Clinical stage	319	​	​	​	​
Stage I&Stage II	90	Reference	​	​	​
Stage III&Stage IV	229	1.298 (0.900–1.870)	0.162	​	​
Pathologic stage	298	​	​	​	​
Stage I&Stage II	70	Reference	​	Reference	​
Stage IV&Stage III	228	2.161 (1.368–3.414)	**< 0.001**	2.172 (1.370–3.445)	**< 0.001**
Histologic grade	321	​	​	​	​
G1&G2	252	Reference	​	​	​
G4&G3	69	1.258 (0.866–1.828)	0.229	​	​
ITGA5	329	​	​	​	​
Low	164	Reference	​	Reference	​
High	165	1.563 (1.129–2.164)	**0.007**	1.541 (1.090–2.176)	**0.014**

Abbreviation: CI, confidence interval; HR, hazard ratio. Events (death): n = 124; Proportional hazards test: all *P* > 0.05.

Bold values indicate statistically significant differences.

**TABLE 2:** Multivariate Cox regression analysis of risk factors for disease specific survival.

Characteristics	Total(N)	Univariate analysis		Multivariate analysis	
		Hazard ratio (95% CI)	P value	Hazard ratio (95% CI)	P value
Gender	312	​	​	​	​
Female	94	Reference	​	​	​
Male	218	1.319 (0.827–2.102)	0.245	​	​
Age	312	​	​	​	​
≤ 60	151	Reference	​	​	​
>60	161	1.197 (0.796–1.802)	0.388	​	​
Clinical stage	302	​	​	​	​
Stage I&Stage II	84	Reference	​	​	​
Stage III&Stage IV	218	1.389 (0.860–2.244)	0.179	​	​
Pathologic stage	281	​	​	​	​
Stage I&Stage II	66	Reference	​	Reference	​
Stage IV&Stage III	215	2.641 (1.395–5.003)	**0.003**	2.528 (1.332–4.799)	**0.005**
Histologic grade	307	​	​	​	​
G1&G2	241	Reference	​	​	​
G4&G3	66	1.323 (0.835–2.095)	0.234	​	​
ITGA5	312	​	​	​	​
Low	158	Reference	​	Reference	​
High	154	1.779 (1.171–2.700)	**0.007**	1.483 (0.958–2.297)	0.077

Abbreviation: CI, confidence interval; HR, hazard ratio. Events (disease-specific death): n = 86; Proportional hazards test: all *P* > 0.05.

Bold values indicate statistically significant differences.

In order to evaluate the potential function of ITGA5 on OSCC progression, we evaluated whether knockdown of ITGA5 affected cell proliferation, migration, and invasion. The Lv-shCon and Lv-shITGA5 lentiviruses were infected into CAL27 and SCC-15 cells. qRT-PCR analysis was used to evaluate the knockdown efficiency, and the results showed that the ITGA5 mRNA expression in the cells infected with Lv-shITGA5 was significantly lower than that in cells infected with Lv-shCon (Figure 3D); we analyzed protein expression of ITGA using Western blot analysis, and found that ITGA5 protein expression in the cells infected with Lv-shITGA5 was significantly lower than that in cells infected with Lv-shCon (Figure 3E), indicating successful ITGA5 knockdown in Lv-shITGA5-infected cells. Then the CCK8 assay and colony formation assay were performed. CCK8 assay showed that cell proliferation was slower in ITGA5-knockdown cells than in control cells, both in CAL27 cells and SCC-15 cells (Figures 3F,G). Clone formation assay showed that, in both CAL27 and SCC-15 cell lines, the number of colonies in ITGA5-knockdown cells was less than that in non-knockdown cells (Figures 3H,I), indicating that ITGA5 knockdown weakened the clone formation capacity of OSCC cells. Wound healing assays were used to evaluate cell migration capacity, and as shown in Figures 3J,K, in the ITGA5 knockdown groups, the wound closure rate was significantly slower in the ITGA5-knockdown groups than in the control groups. In addition, transwell assays were performed to measure cell migration and invasion, as shown in Figures 3L,M, fewer cells in the ITGA5-knockdown groups passed through the transwell chamber than in the control groups. Besides, we performed tumorigenicity assay by injected CAL27 cells with or without ITGA5 knockdown in nude mice. As shown in Figures 3N–P, These results indicate that knockdown of ITGA5 significantly reduced both tumor growth rate and tumor weight. All the above results illustrated that ITGA5 knockdown suppressed OSCC cell proliferation, clone formation, migration, and invasion, as well as tumor growth. In other words, ITGA5 may play an oncogenic role in OSCC.

### 3.4 ITGA5 positively regulated SNAI2 expression

**FIGURE 4:**
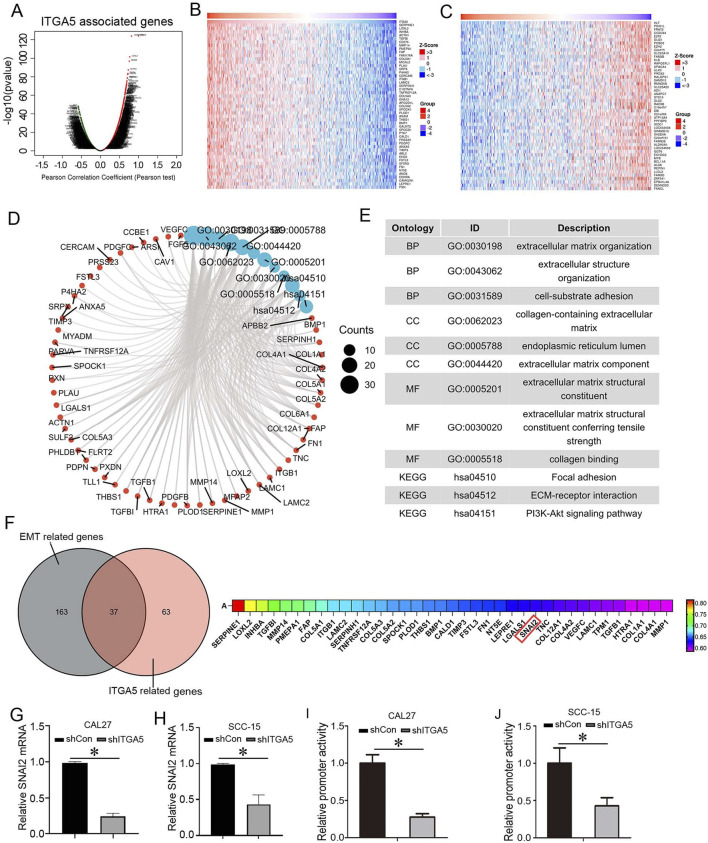
ITGA5 positively regulated SNAI2 expression. **(A)** Volcano plot of genes that are correlated with the expression of ITGA5. **(B)** 50 genes which is most positively correlated with ITGA5. **(C)** 50 genes which is most negatively correlated with ITGA5. **(D,E)** GO analysis and KEGG analysis. **(F)** The intersection of EMT related genes and ITGA5 related genes. **(G,H)** SNAI2 mRNA were detected using qRT-PCR in CAL27 and SCC5 cells with Lv-shCon or Lv-shITGA5 infection. *P < 0.05. **(I,J)** SNAI2 promoter activity were detected using luciferase reporter assay in CAL27 and SCC5 cells with Lv-shCon or Lv-shITGA5 infection. *P < 0.05.

The Linked Omics database was used to screen for genes that are correlated with the expression of ITGA5. As shown in Figure 4A, the volcano plot shows the genes significantly correlated with ITGA5. The 50 genes most positively correlated with ITGA5 were shown in the heatmap in Figure 4B; the 50 genes which is most negatively correlated with ITGA5 were shown in the heat maps in Figure 4C. We conducted GO analysis and KEGG analysis to identify in which functions, characteristics, and signal pathways these genes are enriched (Figure 4D). GO analysis demonstrated that these genes were predominantly enriched in cell-substrate adhesion, extracellular matrix structural constituents and collagen binding, suggesting abnormalities in cell membrane proteins and their association with the EMT process; the KEGG pathway analysis showed that ITGA5 and its related genes were primarily involved in focal adhesion and ECM-receptor interaction pathways (Figure 4E).

When the intersection of EMT-related genes and the top 100 positively related genes of ITGA5 was analyzed, 37 genes were found to be co-expressed (Figure 4F). Among the 37 genes, although SNAI2, also known as SLUG, is not the most highly upregulated gene, but this gene was prioritized for subsequent studies; because of that this gene is a core EMT-activating transcription factor (Stemmler et al., 2019), a biological process tightly linked to ITGA5’s functional enrichment (GO/KEGG analysis identified ITGA5-related genes enriched in cell-substrate adhesion and ECM-receptor interaction pathways, which are critical for EMT). We verified whether SNAI2 is regulated by ITGA5. qRT-PCR analysis showed that SNAI2 mRNA was significantly decreased in cells with Lv-shITGA5 infection compared with these with Lv-shCon infection (Figures 4G,H). Additionally, we designed a SNAI2 promoter-reporter, and transfected the reporter into CAL27 and SCC-15 cells with or without ITGA5 knockdown; as shown in Figures 4I,J, knockdown of ITGA5 induced decrease of SNAI2 promoter activity in these cells. Therefore, SNAI2 was positively regulated by ITGA5 at transcription level.

### 3.5 Overexpression of SNAI2 promoted proliferation, migration, and invasion of OSCC cells

**FIGURE 5:**
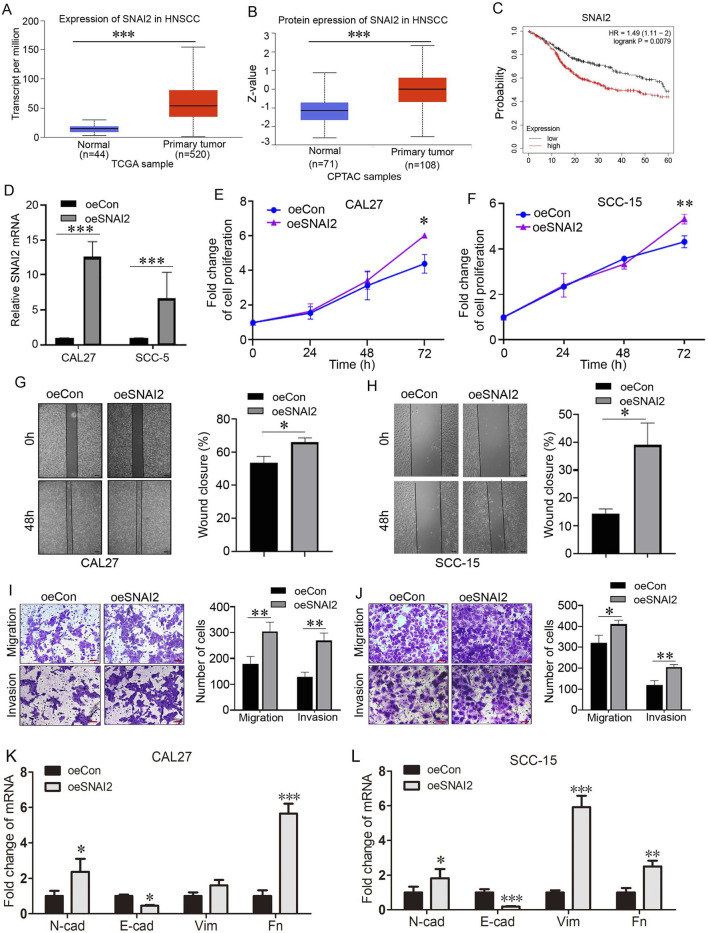
Overexpression of SNAI2 promoted proliferation, migration, and invasion of OSCC cells. **(A)** SNAI2 expression based on TCGA database in normal tissues and primary tumors. ***P < 0.001. **(B)** SNAI2 expression based on CPTAC database in normal tissues and primary tumors. ***P < 0.001. **(C)** Overall survival of HNSCC Patients with low or high SNAI2 expression. **(D)** SNAI2 mRNA was determined using qRT-PCR in CAL27 cells with Lv-oeCon and Lv-oeSNAI2 infection. ***P < 0.001. **(E)** Proliferation of CAL27 cells with Lv-oeCon and Lv-oeSNAI2 infection was detected using CCK-8 assay. *P < 0.05. **(F)** Proliferation of SCC-15 cells with Lv-oeCon and Lv-oeSNAI2 infection was detected using CCK-8 assay. **P < 0.01. **(G)** Wound healing of CAL27 cells with Lv-oeCon and Lv-oeSNAI2 infection. Percentage of wound closure (right panel). *P < 0.05. Bar = 50 μm. **(H)** Wound healing of SCC-15 cells with Lv-oeCon and Lv-oeSNAI2 infection. Percentage of wound closure (right panel). *P < 0.05. Bar = 100 μm. **(I)** Cell migration and invasion of CAL27 cells with Lv-oeCon and Lv-oeSNAI2 infection weredetected by transwell assay. Numbers of cells passing through the transwell chambers (right panel). **P < 0.01. Bar = 50 μm. **(J)** Cell migration and invasion of SCC-15 cells with Lv-oeCon and Lv-oeSNAI2 infection weredetected by transwell assay. Numbers of cells passing through the transwell chambers (right panel). *P < 0.05, **P < 0.01. Bar = 50 μm. **(K,L)** E-cadherin, N-cadherin, Vimentin, Fibronectin 1 mRNA was determined using qRT-PCR in CAL27 cells and SCC-15 cells. *P < 0.05, **P < 0.01, ***P < 0.001 vs. oeCon. E-cad: E-cadherin; N-cad: N-cadherin; VIM: Vimentin; Fn: Fibronectin 1.

We analyzed SNAI2 expression based on TCGA database, and as shown in Figure 5A, in primary tumor, more SNAI2 transcripts were detected than in normal tissues, indicating that SNAI2 was overexpressed in HNSCC. Based on CPTAC database information, we further analyzed the protein of SNAI2, and as shown in Figure 5B, compared with normal tissues, the SNAI2 protein is highly expressed in HNSCC compared with normal tissues. In addition, HNSCC patients with high expression of SNAI2 possess poor prognosis compared with patients with low SNAI2 expression (Figure 5C). Multivariate Cox regression analysis (Table 3) was conducted to assess prognostic factors for survival among relevant patients. Pathologic N stage N2&N3 was an independent poor prognostic factor (HR = 1.985, 95% CI:1.401–2.812, *P* < 0.001). Pathologic T3 (HR = 1.919, *P* = 0.082) and T4 (HR = 1.840, *P* = 0.091) stages approached significance. N1 stage (*P* = 0.997), T2 stage (*P* = 0.987), and high SNAI2 expression (*P* = 0.231) showed no significant associations with survival. Univariate analysis confirmed T3 (HR = 2.592, *P* = 0.004), T4 (HR = 2.347, *P* = 0.008), and N2&N3 (HR = 2.269, *P* < 0.001) as risk factors, while SNAI2 expression was not significant. To explore the role of SNAI2 in OSCC, we constructed cells with SNAI2 overexpression by lentivirus infecting in CAL27 and SCC-15 cell lines, and analyzed cell proliferation, migration and invasion. As shown in Figure 5D, SNAI2 mRNA levels were elevated in Lv-oeSNAI2-infected cells, indicating that SNAI2 was overexpressed. CCK-8 assay was used to measure the proliferation ability, and as shown in Figures 5E,F, cell proliferation was promoted in cells with SNAI2 overexpression. In addition, wound healing assay showed that overexpression of SNAI2 induced significant elevation of healing closure rate, indicating the positive role of SNAI2 in cell migration (Figures 5G,H). Moreover, Transwell migration and invasion assays were performed. As shown in Figures 5I,J, both migration and invasion assay indicated that the number of cells that passed through the membrane in the SNAI2 overexpression group was significantly higher than that in the control group, that is to say SNAI2 promoted cell migration and invasion. We measuring several canonical markers using RT-qPCR. The results showed that in CAL27 cells, overexpression of SNAI2 induced downregulation of E-cadherin, as well as upregulation of N-cadherin and Fibronectin; however, overexpression of SNAI2 induced a slight increase of Vimentin mRNA (with no significance) (Figure 5K). The results showed that in SCC-15 cells, overexpression of SNAI2 induced downregulation of E-cadherin, as well as upregulation of N-cadherin, Vimentin and Fibronectin (Figure 5L). Therefore overexpression of SNAI2 enhanced EMT in these two cells. In summary, SNAI2 exerts pro-tumor effects in OSCC cells.

**TABLE 3:** Multivariate Cox regression analysis of risk factors for disease specific survival.

Characteristics	Total(N)	Univariate analysis		Multivariate analysis	
		Hazard ratio (95% CI)	P value	Hazard ratio (95% CI)	P value
Pathologic T stage	447	​	​	​	​
T1	45	Reference	​	Reference	​
T2	134	1.341 (0.694–2.590)	0.382	1.006 (0.475–2.131)	0.987
T3	96	2.592 (1.349–4.984)	**0.004**	1.919 (0.921–3.999)	0.082
T4	172	2.347 (1.250–4.407)	**0.008**	1.840 (0.906–3.733)	0.091
Pathologic N stage	410	​	​	​	​
N0	170	Reference	​	Reference	​
N1	66	0.957 (0.563–1.624)	0.870	1.001 (0.589–1.703)	0.997
N2&N3	174	2.269 (1.617–3.183)	**< 0.001**	1.985 (1.401–2.812)	**< 0.001**
SNAI2	503	​	​	​	​
Low	251	Reference	​	​	​
High	252	1.177 (0.902–1.537)	0.231	​	​

Bold values indicate statistically significant differences.

### 3.6 ITGA5 promoted proliferation, clone formation, migration and invasion of OSCC cells through SNAI2

**FIGURE 6:**
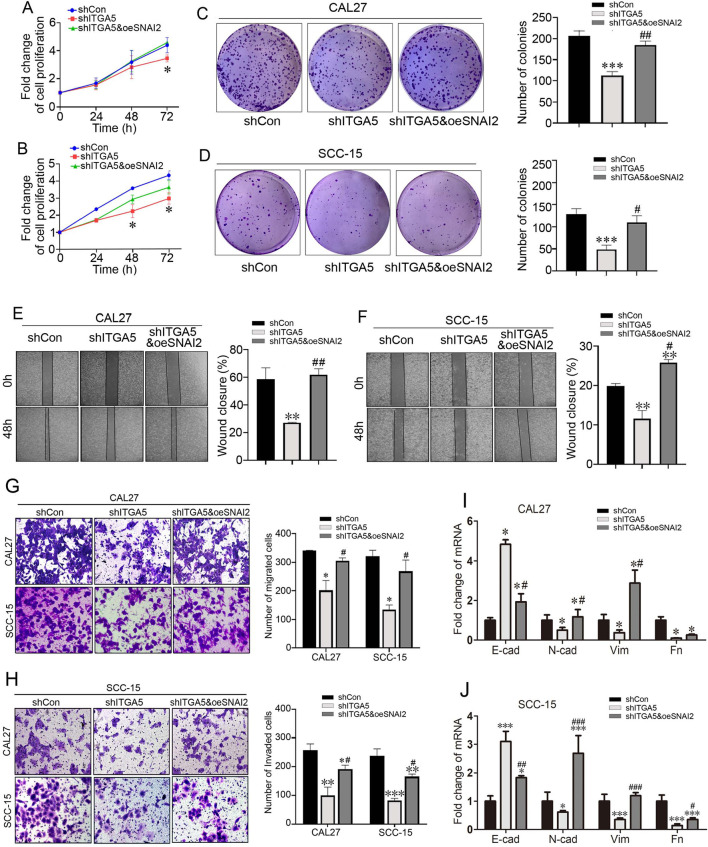
ITGA5 promoted proliferation, clone formation, migration and invasion of OSCC cells through SNAI2. **(A)** Proliferation of CAL27 cells infected with Lv-shCon, Lv-shITGA5 and Lv-shITGA5&Lv-oeSNAI2 determined with CCK8 assay. *P < 0.05. **(B)** Proliferation of SCC-15 cells infected with Lv-shCon, Lv-shITGA5 and Lv-shITGA5&Lv-oeSNAI2 determined with CCK8 assay. *P < 0.05. **(C)** Clone formation of CAL27 cells infected with Lv-shCon, Lv-shITGA5 and Lv-shITGA5&Lv-oeSNAI2. Number of cell colonies (right panel). ***P < 0.001 vs. shCon; ^##^ *P* < 0.01 vs. shITGA5. **(D)** Clone formation of SCC-15 cells infected with Lv-shCon, Lv-shITGA5 and Lv-shITGA5&Lv-oeSNAI2. Number of cell colonies (right panel). ***P < 0.001 vs. shCon; ^#^ *P* < 0.05 vs. shITGA5. **(E)** Wound healing of CAL27 cells infected with Lv-shCon, Lv-shITGA5 and Lv-shITGA5&Lv-oeSNAI2. The percentage of wound closure (right panel). **P < 0.01 vs. shCon, ^##^ *P* < 0.01 vs. shITGA5. Bar = 10 μm. **(F)** Wound healing of CAL27 cells infected with Lv-shCon, Lv-shITGA5 and Lv-shITGA5&Lv-oeSNAI2. The percentage of wound closure (right panel). **P < 0.01 vs. shCon, ^#^ *P* < 0.05 vs. shITGA5. Bar = 10 μm. **(G)** Migration of CAL27 cells and SCC-15 cells infected with Lv-shCon, Lv-shITGA5 and Lv-shITGA5&Lv-oeSNAI2 was determined using Transwell assay. Number of migrated cells (lower panel). *P < 0.05 vs. shCon; ^#^ *P* < 0.05 vs. shITGA5. Bar = 50 μm. **(H)** Invasion of CAL27 cells and SCC-15 cells infected with Lv-shCon, Lv-shITGA5 and Lv-shITGA5&Lv-oeSNAI2 was determined using Transwell assay. Number of invaded cells (lower panel). *P < 0.05, **P < 0.01, **P < 0.05 vs. shCon; ^#^ *P* < 0.05 vs. shITGA5. Bar = 50 μm. **(I,J)** E-cadherin, N-cadherin, Vimentin, Fibronectin 1 mRNA was determined using qRT-PCR in CAL27 cells and SCC-15 cells. *P < 0.05, **P < 0.01 vs. shCon; ^#^ *P* < 0.05, ^##^ *P* < 0.01, ^###^ *P* < 0.001 vs. shITGA. E-cad: E-cadherin; N-cad: N-cadherin; VIM: Vimentin; Fn: Fibronectin 1.

To determine whether the oncogenic effects of ITGA5 depend on SNAI2, we overexpressed SNAI2 in Lv-shITGA5 cells (CAL27 and SCC-15) and detected cell proliferation, clone formation, cell migration and invasion capacities. CCK8 assay showed that overexpression of SNAI2 reversed the inhibition of cell proliferation induced by ITGA5 knockdown (Figures 6A,B). The decreased clone formation ability induced by ITGA5 knockdown was also partially reversed by overexpression of SNAI2 (Figures 6C,D). Furthermore, using wound healing experiment, we observed that the inhibitory effect of ITGA5 knockdown on cell migration of OSCC cells was blocked by SNAI2 upregulation (Figures 6E,F). In addition, Transwell migration and invasion assays were employed to assess the migration and invasion abilities of cells. Both cell migration (Figure 6G) and invasion (Figure 6H) were partially antagonized by SNAI2 upregulation. We measuring several canonical markers using RT-qPCR. The results showed that in CAL27 cells, knockdown of ITGA5 induced upregulation of E-cadherin, as well as downregulation of N-cadherin, Vimentin and Fibronectin; however, the regulation of E-cadherin, N-cadherin, and Vimentin were reversed by overexpression of SNAI2 (Figure 6I). In SCC-15 cells, knockdown of ITGA5 induced upregulation of E-cadherin, as well as downregulation of N-cadherin, Vimentin and Fibronectin; however, the regulation of E-cadherin, N-cadherin, and Fibronectin were reversed by overexpression of SNAI2 (Figure 6J). Therefore overexpression of SNAI2 reversed konckdown of ITGA5 induced EMT inhibition in these two cells. These results suggested that ITGA5 facilitates proliferation, colony formation, migration and invasion of OSCC cells through SNAI2.

## 4 Discussion

OSCC is characterized by high local recurrence and poor prognosis (Li et al., 2017). Therefore, it is crucial to investigate the mechanisms underlying the initiation and progression of OSCC, as well as to implement strategies for early diagnosis, prevention, and treatment to reduce the risk of recurrence. In this study, we demonstrated that ITGA5 was significantly elevated in OSCC, and that ITGA5 regulated OSCC cell proliferation, migration, and invasion through SNAI2.

The evolving landscape of molecular biology and genetic research, combined with advancements in molecular diagnostics and immunohistochemical techniques, has significantly accelerated the discovery of molecular biomarkers for OSCC proliferation, diagnosis, and prognosis (Pekarek et al., 2023; Rengasamy et al., 2025; Yang P. et al., 2025). Among these biomarkers, the integrin family has emerged as a crucial focus in OSCC research (Santosh, Jones and Harvey, 2016). Integrins represent a class of transmembrane proteins that mediate biological functions, such as cell survival, cell proliferation, cell migration, and invasion, by regulating cytoskeletal reorganization and activating intracellular signaling pathways (Cooper and Giancotti, 2019; Slack et al., 2022). ITGA5 is an important member of the integrin family, serving as a crucial bridge connecting the extracellular matrix and intracellular signal transduction pathways, and correspondingly participates in various essential physiological processes, such as tumor invasion and metastasis (Ka et al., 2025; Yao et al., 2023). We and other researchers have proved ITGA5 can serve as a prognostic biomarker for HNSCC (Feng et al., 2020; Liu et al., 2024; Zou et al., 2019). As OSCC is a main subtype of HNSCC, in this study, we identified the role of ITGA5 in OSCC. We analyzed ITGA5 expression in OSCC and found that ITGA5 was overexpressed in OSCC compared with normal tissues; in addition, we performed *in vitro* experiments and demonstrated that downregulation of ITGA5 resulted in inhibition of cell proliferation, colony formation, as well as cell migration and invasion. Our findings of ITGA5 promoting cell migration and invasion-key early metastatic steps, suggest it may facilitate metastasis formation. The ITGA5-driven invasive phenotype could aid tumor cell entry into the bloodstream, survival in circulation by resisting anoikis through fibronectin adhesion, and exit at distant sites. ITGA5’s role in ECM signaling may further support micrometastasis outgrowth. Its correlation with poor patient prognosis aligns with a pro-metastatic role, warranting future validation *in vivo*. Our finding is consistent with a previous study (Fan et al., 2019), in which the authors found that ITGA5 was overexpressed in OSCC tissues, knockdown of ITGA5 inhibited cell proliferation, migration, and invasion; however, regarding the mechanism, they only demonstrated that knockdown of ITGA5 can inhibit the PI3K/AKT signaling pathway, but whether PI3K/AKT plays a major role in the regulation of OSCC progression by ITGA5 has not been investigated. Compared with the above study, we have demonstrated that ITGA5 affects the progression of OSCC through SNAI2, which updates the understanding of the mechanism by which ITGA5 regulates OSCC progression and provides a theoretical basis for developing methods for OSCC diagnosis and treatment based on ITGA5. A minor limitation of this study is that only one shRNA sequence was used for ITGA5 knockdown. Although the knockdown efficiency was validated at both mRNA and protein levels, and consistent phenotypic changes were observed in two independent OSCC cell lines, future studies will employ a second independent shRNA to further confirm the specificity of ITGA5 function and rule out potential off-target effects.

SNAI2, a well-recognized transcriptional inhibitor (Nieto, 2002), is involved in epithelial-mesenchymal transition (EMT) and exhibits tumor-promoting effects by binding to E-box motifs (Deng et al., 2024; Fan et al., 2020). Additionally, SNAI2 plays important roles in the progression of various tumors: SNAI2 significantly promotes the dormancy of HPV-negative cervical cancer cells by modulating the expression of urokinase-type plasminogen activator receptor (u-PAR) and fine-tuning the activity of the ERK/p38 signaling pathway (Zhou et al., 2024); SNAI2 promotes the migration and invasion of ovarian cancer cells through regulating ferroptosis (Jin et al., 2022). SNAI2 correlates with poor prognosis of patients with HNSCC (Wan et al., 2020). In this study, through bioinformatics analysis, we also confirmed that SNAI2 was overexpressed in HNSCC and high SNAI2 expression was associated with poor outcomes of patients with this disease. More importantly, we overexpressed SNAI2 in two OSCC cell lines, and demonstrated the promotional roles of SNAI2 in cell proliferation, cell migration, and invasion, indicating its oncogenic properties in OSCC. However, the mechanism by which SNAI2 influences the progression of OSCC requires further investigation. As a transcription factor, SNAI2 participates in the development of various tumors through regulating several downstream target genes, for example, SNAI2 transcriptionally activated FTH1P3 expression and correspondingly promoted colorectal cancer metastasis (Deng et al., 2024). Therefore, we speculate that SNAI2 participates in OSCC progression through a similar mechanism, and transcriptome sequencing in OSCC cells with SNAI2 overexpression or knockdown can be used to identify the downstream genes of SNAI2.

In this study, we identified a potential association between ITGA5 and SNAI2 using bioinformatics analysis, and then verified that knockdown of ITGA5 induced downregulation of SNAI2; besides, we performed a luciferase promoter-reporter assay and found that knockdown of ITGA5 significantly decreased the promoter activity of SNAI2 in OSCC cells, providing direct mechanistic evidence supporting that ITGA5 functions upstream of SNAI2 at the transcriptional level in OSCC. We found that overexpression of SNAI2 reversed the ITGA5-knockdown-induced decrease of cell proliferation, colony formation, cell migration and invasion, therefore, SNAI2 participate in ITGA5-regulated OSCC progression. A limitation that we have not performed SNAI2 knockdown experiments in ITGA5-overexpressing cells to further verify the necessity of SNAI2 in mediating ITGA5-driven cellular phenotypes. We propose that future studies will address this gap by conducting reciprocal rescue experiments to fully clarify the functional hierarchy and molecular crosstalk between ITGA5 and SNAI2 in OSCC progression. A previous study revealed that ITGA5 promotes the progression of OSCC through the PI3K/AKT signaling pathway (Fan et al., 2020); whether SNAI2 and PI3K/AKT signaling pathway interact or two independent mechanisms in ITGA5 regulating OSCC is worthy of further exploration. Beyond our findings, other factors can affect OSCC progression through regulating SNAI2: FOXD1 promotes EMT and cell stemness in OSCC by regulating SNAI2 transcription (Chen et al., 2021); TGF-β1 elevated the expression of SNAI2 through ERK1/2 and correspondingly promoted cell migration and invasion (Joseph et al., 2009). These findings and our study reflect that SNAI2 may be a candidate hub for several factors or signaling pathways in the progression of OSCC; therefore, in anti-OSCC therapy, targeting SNAI2 may exert a synergistic effect on multiple upstream factors or pathways.

SNAI2 is regulated by various factors at different levels, such as miRNA in post-transcriptional level (Feng et al., 2024; Li et al., 2024), as well as in post-translational modification level affecting protein stability (He et al., 2024; Qiao et al., 2023). One limitation of this study is that we found that ITGA5 knock-down lowers SNAI2 levels and that SNAI2 rescue reverses the phenotype, but this does not demonstrate how ITGA5 regulates SNAI2. To date, we do not know the mechanism by which ITGA5 regulates SNAI2 in OSCC. Gene expression can occur at different levels, such as at the transcriptional level, in post-transcriptional level, in translational level, and through protein post-translation. Considering that SNAI2 mRNA was altered, we proposed that ITGA5 regulates SNAI2 at the transcriptional level or in post-transcriptional level. Future research can focus on whether ITGA5 can regulate the promoter activity of SNAI2 and identify the putative transcription factor that mediates this regulation, or investigate whether ITGA5 influences the stability of SNAI2 mRNA through any miRNA. Besides, there are evidences about kinases regulating transcriptional factors: Polo-like kinase 1 (PLK1), one of the kinases, involved in arsenite-induced NOTCH1 down-modulation during DNA damage and mitoticprogression, supporting that the effect of ITGA5 regulating SNAI2 could be mediated via kinase signaling or cell cycle checkpoints (De Blasio et al., 2019). ITGA5 has been reported associated with FAK/ERK (Liu et al., 2022; Yao et al., 2020) and PI3K/AKT (Shi et al., 2025) signal pathways, which are pathways regulate SNAI2 (Park et al., 2024; Yang P. W. et al., 2025), supporting the plausibility of FAK/ERK/AKT mediating the ITGA5-SNAI2 regulatory axis. Similar regulatory balances between proliferation and apoptosis have been described in keratinocyte models where ASK1 and p21 determine cell fate under stress, supporting the idea that ITGA5 may influence survival pathways in OSCC cells (De Blasio et al., 2021). Future investigations will focus on validating these pathway readouts and inhibitor-based experiments to establish a definitive causal mechanism.

Other limitations: we analyzed the expression and significance of ITGA5 in OSCC using bioinformatics methods based on open access data such as TCGA; in fact, collecting OSCC specimens from clinical settings, especially from multicenter specimens, and detecting ITGA5 expression to verify its correlation with patients’ clinicopathological characteristics can provide a more solid basis for the application of ITGA5 in the diagnosis and treatment of OSCC. Furthermore, Consistent with our *in vitro* observations, subcutaneous xenograft tumor models further demonstrated that knockdown of ITGA5 significantly suppressed tumor growth *in vivo*, supporting the oncogenic role of ITGA5 in OSCC. These findings provide preliminary *in vivo* evidence reinforcing the functional importance of ITGA5 in OSCC progression. Nevertheless, this study still possesses certain limitations regarding *in vivo* validation. Although the inhibitory effect of ITGA5 knockdown on tumor growth was confirmed in subcutaneous xenografts, more sophisticated *in vivo* investigations, including orthotopic tumor models, distant metastasis assays, and *in vivo* rescue experiments involving SNAI2 restoration, remain to be performed. Therefore, further *in vivo* validation is warranted to consolidate the therapeutic potential of targeting the ITGA5/SNAI2 axis in OSCC. Besides, as HPV/p16 status-a critical prognostic factor in OSCC was not analyzed, the relevance of the ITGA5-SNAI2 axis specifically in the HPV/p16-positive minority (∼5–15%) remains unknown; the observed link between high ITGA5 and poor prognosis requires validation in models adjusted for established covariates like tumor stage, nodal status, and tobacco/alcohol use. Future work must stratify by HPV/p16 status and, crucially, employ large multi-center cohorts with comprehensive clinical data to validate ITGA5’s independent prognostic value via multivariate analysis. Integrating such clinical data with bioinformatics from public databases will be essential for translating this mechanistic insight into precise clinical strategies for OSCC. Additionally, we explored the relationship between ITGA5 and HNSCC; therefore, investigating the significance of ITGA5 in the progression of other HNSCC subtypes, such as laryngeal cancer and oropharyngeal cancer, is necessary and of great significance.

## 5 Conclusion

In summary, we conducted a comprehensive assessment of ITGA5, providing preliminary evidence for its potential role in OSCC progression and as a candidate indicator for prognosis and therapy. Our findings indicate that the ITGA5-SNAI2 pathway is functionally involved in OSCC malignant progression, supporting its potential as a prognostic candidate and therapeutic target. However, it is important to note that these claims remain preliminary and require further validation-including large-scale OSCC-specific cohort studies with survival analyses adjusted for key clinical covariates (e.g., tumor stage, nodal status, age, treatment, and tobacco/alcohol exposure) and *in vivo* validation using orthotopic growth/invasion or experimental metastasis models. Future studies addressing these aspects will help consolidate the translational value of the ITGA5-SNAI2 pathway for OSCC prognosis and targeted therapy.

## Data Availability

The datasets presented in this study can be found in online repositories. The names of the repository/repositories and accession number(s) can be found below: 10.6084/m9.figshare.31329751.
