# Supplementary material for: ITGA5 is overexpressed and promotes tumor progression through SNAI2 in OSCC
Source: Front Cell Dev Biol. 2026 May 21;14:1685462. doi: 10.3389/fcell.2026.1685462 (PMC13233712; doi:10.3389/fcell.2026.1685462)
Supplement: Supplementary file 1 [file Table1.docx]

| Gene | Forward Primer (5’-3’) | Reverse Primer (5’-3’) | amplicon lengths (bp) |
| --- | --- | --- | --- |
| ITGA5 | GCCTGTGGAGTACAAGTCCTT | AATTCGGGTGAAGTTATCTGTGG | 163 |
| SNAI2 | ATCTGCGGCAAGGCGTTTTCCA | GAGCCCTCAGATTTGACCTGTC | 127 |
| E-cadherin | GCTGGACCGAGAGAGTTTCC | CAAAATCCAAGCCCGTGGTG | 338 |
| N-cadherin | TGGACCATCACTCGGCTTA | ACACTGGCAAACCTTCACG | 156 |
| Vimentin | GCAGGAGGCAGAAGAATGGT | CCACTTCACAGGTGAGGGAC | 140 |
| Fibronectin 1 | GCAGCATGACATGGGTCACA | CATCAACAATGCACTGATCTCG | 107 |
| β-actin | CATGGAGTCCTGTGGCATC | CAGGGCAGTGATCTCCTTCT | 157 |

Supplementary Table 1. primers for qRT-PCR.
